# A shape-independent analytical method for gear mesh stiffness with asymmetric spalling defects

**DOI:** 10.1038/s41598-024-66511-1

**Published:** 2024-07-05

**Authors:** Yi Jin, Qingyuan Zhang, Yunxia Chen, Tianpei Zu

**Affiliations:** 1 School of Intelligent Manufacture, Taizhou University, Jiaojiang, 318000 China; 2 Hangzhou International Innovation Institute, Beihang University, Hangzhou, 311115 China; 3 School of Reliability and System Engineering, Beihang University, Beijing, 100191 China; 4 School of Aeronautic Science and Engineering, Beihang University, Beijing, 100191 China; 5 Science and Technology on Reliability and Environmental Engineering Laboratory, Beihang University, Beijing, 100191 China

**Keywords:** Energy infrastructure, Mechanical engineering

## Abstract

Spalling, a common failure mechanism of gear systems, greatly affects the dynamics of gears operation, which is reflected in the time-varying mesh stiffness (TVMS). Current TVMS models often overestimate the asymmetric spalling phenomenon and may lead to inaccuracy in identifying and predicting the spalling failure. To address this problem, in this paper, a new stiffness, namely torsional stiffness, is introduced to quantify the effect of asymmetric spalling defects, and an equivalent stiffness calculation method for different asymmetric shapes is proposed. Based on this, a shape-independent TVMS model is constructed, which can realize the fast calculation of TVMS for spalling defects with different shapes at arbitrary asymmetric locations. Furthermore, a FEM-based validation method is developed by considering diverse loading states and improving the current result extraction method. Case studies are presented to illustrate the proposed model and to analyze the effects of different types of asymmetric spalling defects on gear dynamics. The FEM validation has shown that the proposed model has a good effectiveness.

## Introduction

Gear systems are typical transmission elements in power equipment, such as aircraft engines, wind turbines, and marine diesel engines, which operate under severe conditions of high speed, heavy load, and poor lubrication.Under these conditions, tooth spalling often occurs in the gear system, leading to a change in the dynamics and shortening the service life of the gear^1,2^. The time-varying mesh stiffness (TVMS), as the critical excitation source of the gear system, is a crucial factor in modelling gear dynamics. It directly reflects the impact of spalling defects on the dynamic response^3^. Therefore, an accurate and quick evaluation of gear TVMS in the case of tooth spalling is essential to reveal the dynamic behaviour and develop failure prevention measures of the gear system.

Numerous calculation methods have been developed to investigate the TVMS of the spalled gear pairs. These methods can be classified into four categories: experiments^4–6^, finite element method (FEM)^7–9^, analytical method^10,11^ and analytical-FE method^12,13^. Experimentation is the most realistic and reliable method for assessing stiffness but is rarely used due to the need for specialised and expensive monitoring equipment^11^. FEM can create a numerical model of the meshing gear pair instead of physical experiments and can achieve accurate TVMS calculations at the expense of computational time. Analytical-FE method adopts the Hertzian contact theory to calculate local contact deformation and still uses FEM as a basis to calculate tooth deflections of total gears. As a result, it has a certain level reduction of computation but is still time-consuming^14^. Compared with FEM and analytical-FE method, analytical method can theoretically establish the relationship among gear parameters, spalling defects and stiffness, which is superior in higher computational efficiency but may suffer from certain calculation errors.

Many researchers have carried out a series of derivations and corrections of the TVMS model under various types of spalling defects, aiming to reduce calculation errors. The most common analytical method found in the literature is the potential energy method (PEM)^15^. At first, a series of PEMs have been proposed for regular spalling patterns, such as rectangular spallings^16,17^, circular spallings^18^ and V-shaped spallings^19^, with constant spalling depth. And all these models performed well in finite element validation. Nevertheless, the assumption of constant spalling depth still has some differences compared to the actual morphological characteristics of regular spalling. In this regard, Luo et al.^20^ and Cheng et al.^21^ adopted an elliptical geometry to describe the spalling defects, which is able to change the radius in three dimensions to better match the actual spalling morphology. Further, a TVMS model for elliptical geometry using the potential energy method was derived and better results were obtained. Compared with regular-shaped spalling, irregular-shaped spalling is more common in practice. However, the models presented above have difficulty in covering irregular spallings^22^. Thus, some scholars have proposed modified models to overcome this issue. Yousfi et al.^23^ proposed a double discretization of the gear tooth surface to account for irregular defect depth variations in the width and length directions of the gear tooth. They also developed a contact detection algorithm to identify the actual contact points during meshing. Wu et al.^24^ used a polynomial approximation to fit a series of spalling length data to irregular spalling profiles. This approximation was then substituted into the PEM to directly calculate the TVMS. Thus, the method requires a series of tooth parameter transformations to accurately calculate the TVMS under irregular spalling, which is not convenient to implement due to the need to identify tooth profile variations on individual slices of the gear. To describe the effect of irregular spalling on the cross-sectional parameters, Luo et al.^25^ introduced defect ratios, which can equate the irregular spalling cross-section parameters to those of a normal rectangle. Based on this, a generalized TVMS model for spalled gear teeth was developed, which has good applicability since only the geometric features of the spalling defects need to be extracted.

**Figure 1:**
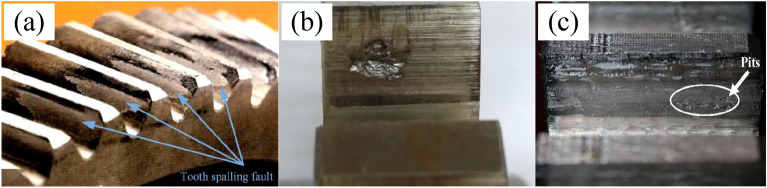
Actual asymmetric spalling defect^22,26,27^.

In summary, the above literature review presents a series of well-validated TVMS models proposed for spalling defects with different shapes. However, the aforementioned models based on the symmetry assumption of spalling defects do not accurately represent the effect of asymmetric spalling on TVMS. In practice, it is a frequent occurrence that the spalling deviates from the center section, as depicted in Fig. 1. The existing models derived from the potential energy method do not consider the impact of the spalling offset. As a result, the calculated meshing stiffnesses for the same spalling defects at different offsets are identical. This is obviously inconsistent with the actual meshing response. In literature, Saxena et al.^19^ once provided a brief remark about the impact of torque resulting from asymmetric rectangular spalling, but unfortunately, its specific effect on the TVMS were not discussed in detail. Thus, the accurate and efficient calculation of various types of asymmetric spalling on TVMS is still a challenging problem.

To handle the above problems, this paper aims to enhance the current TVMS analytical models by introducing a new sub-stiffness to represent the asymmetric effect. Based on this, a generic TVMS model capable of describing any spalling shape and location and its simulation verification method are proposed, allowing the effect of spalling on TVMS to be rapidly evaluated. The main technical contributions of this paper are summarized as follows:

- A new sub-stiffness, called torsional stiffness, is derived. This stiffness considers the effects of non-uniform loading caused by asymmetric spalling and makes the TVMS results more consistent with the actual meshing characteristics.
- A shape-independent TVMS model is proposed using the cross-sectional equivalence method. This model is capable of quickly calculating the TVMS of spalling defects with various shapes at arbitrary asymmetric locations.
- The finite element verification method has been refined to account for the uneven angular displacement characteristics of the gear inner ring, which are caused by the positional distribution of spalling defects. This refinement facilitates more reflective evaluation of the influence of the spalling location.

The rest of this article is organized as follows: The second section provides a brief description of the basic spalled mesh stiffness model. Additionally, a torsional stiffness model is incorporated into the basic mesh stiffness model to characterise the effects of asymmetric spalling. An equivalent calculation method for torsional stiffness is proposed in “Shape-independent TVMS evaluation” section. A generic TVMS model is then constructed to evaluate the mesh stiffness under different asymmetric spalling defects. In “Simualtion validation method for asymmetric spalling” section, a FEM-based validation method is proposed to better describe the difference of the spalling location on the TVMS. The applicability of the proposed model for regular shapes (rectangular, elliptical) and irregular shape is further discussed in “Results and discussion” section, and the FEM is constructed for different spalling locations for comparison. Finally, “Conclusion” section concludes this article.

## An improved mesh stiffness model with asymmetric spalling

Asymmetric spalling can frequently arise during gear operation, attributable to the random distribution of defects and load uncertainty. The existing TVMS models, which are basically based on symmetry assumption, cannot describe the asymmetric spalling effect accurately. To provide a precise TVMS model, this chapter first provide a basic mesh stiffness model for spalling. Then, we discuss the effects of asymmetric spalling defects in detail and present a novel type of stiffness called torsional stiffness to describe the magnitude of spalling offset.

### Basic mesh stiffness model for spalling

**Figure 2:**
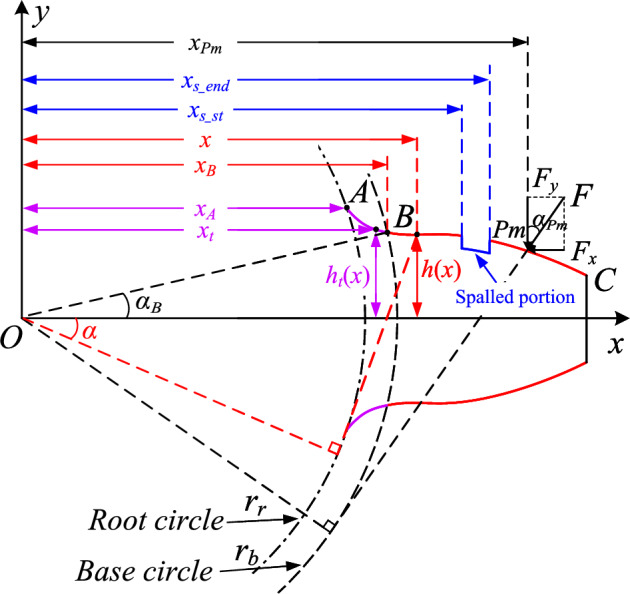
Gear tooth modeled as a cantilever beam.

To model the meshing gear system, a gear tooth is usually simplified as a cantilever beam of variable cross-section on the root circle, as shown in Fig. 2. According to the potential energy method, the total potential energy $U_{total}$ stored in the meshing gear system include five parts, i.e. Hertzian contact energy $U_h$, axial compressive energy $U_a$, bending energy $U_b$, shear energy $U_s$ and fillet foundation energy $U_f$, and can be calculated as:

1 $$\begin{aligned} \begin{aligned} U_{total}&=\dfrac{F^2}{2k_{total}}=\sum \limits _{i=1}\limits ^n{\left( U_{ag,i}+U_{bg,i}+U_{sg,i}+U_{fg,i}+U_{ap,i}+U_{bp,i}+U_{sp,i}+U_{fp,i}+U_{h,i} \right) }\\&=\dfrac{F^2}{2}\sum \limits _{i=1}\limits ^n{\left( \dfrac{1}{k_{ag,i}}+\dfrac{1}{k_{bg,i}}+\dfrac{1}{k_{sg,i}}+\dfrac{1}{k_{fg,i}}+\dfrac{1}{k_{ap,i}}+\dfrac{1}{k_{bp,i}}+\dfrac{1}{k_{sp,i}}+\dfrac{1}{k_{fp,i}}+\dfrac{1}{k_{h,i}} \right) }, \end{aligned} \end{aligned}$$

where *F* is the contact force, *n* represents the number of tooth pairs in meshing at the same time, the subscript *p*, *g* stand for pinion and gear, respectively. $k_{a}$, $k_{b}$, $k_{s}$, $k_{f}$, and $k_{h}$ are the corresponding axial compressive stiffness, bending stiffness, shear stiffness, fillet foundation stiffness and Hertzian contact stiffness.

For $k_a$, $k_b$ and $k_s$, the dominant method is to calculate the axial compressive, bending and shear stiffness consisting of the transitional portion, healthy and spalled involute portions by segmental integration. In addition, considering that the mesh point *Pm* is in different regions of the involute curve as the gear rotates, the aforementioned sub-stiffnesses can be expressed by a segmented function, namely:

2 $$\begin{aligned} \frac{1}{k_{a,i}}\!= & {} \!{\left\{ \begin{array}{ll} \displaystyle { \int _{x_A}^{x_B}{\dfrac{\sin ^2\alpha _{P_m}}{EA_t\left( x_t \right) }dx_t} \!+\!\int _{x_B}^{x_{Pm}}{\dfrac{\sin ^2\alpha _{P_m}}{EA\left( x \right) }dx}},&{} x_B<{ x_{Pm}}\leqslant x_{s\_st}\\ \displaystyle { \int _{x_A}^{x_B}{\dfrac{\sin ^2\alpha _{P_m}}{EA_t\left( x_t \right) }dx_t}\!+\!\int _{x_B}^{x_{s\_st}}{\dfrac{\sin ^2\alpha _{P_m}}{EA\left( x \right) }dx}\!+\!\int _{x_{s\_st}}^{x_{Pm}}{\dfrac{\sin ^2\alpha _{P_m}}{EA_s\left( x \right) }dx}},&{} x_{s\_st}<{ x_{Pm}}\leqslant x_{s\_end}\\ \displaystyle { \int _{x_A}^{x_B}{\dfrac{\sin ^2\alpha _{P_m}}{EA_t\left( x_t \right) }dx_t}\!+\!\int _{x_B}^{x_{s\_st}}{\dfrac{\sin ^2\alpha _{P_m}}{EA\left( x \right) }dx}\!+\!\int _{x_{s\_st}}^{x_{s\_end}}{\dfrac{\sin ^2\alpha _{P_m}}{EA_s\left( x \right) }dx}}\\ \displaystyle { \!+\!\int _{x_{s\_end}}^{x_{P_m}}{\dfrac{\sin ^2\alpha _{P_m}}{EA\left( x \right) }dx}}.&{} x_{s\_end}<{ x_{Pm}}\\ \end{array}\right. } \end{aligned}$$

3 $$\begin{aligned} \frac{1}{k_{b,i}}\!= & {} \!{\left\{ \begin{array}{ll} \displaystyle { \int _{x_A}^{x_B}{\dfrac{f\left( x_t \right) }{EI_{zt}\left( x_t \right) }dx_t}\!+\!\int _{x_B}^{x_{P_m}}{\dfrac{f\left( x \right) }{EI_z\left( x \right) }dx}},&{} x_B<{ x_{Pm}}\leqslant x_{s\_st}\\ \displaystyle { \int _{x_A}^{x_B}{\dfrac{f\left( x_t \right) }{EI_{zt}\left( x_t \right) }dx_t}\!+\!\int _{x_B}^{x_{s\_st}}{\dfrac{f\left( x \right) }{EI_z\left( x \right) }dx}\!+\!\int _{x_{s\_st}}^{x_{P_m}}{\dfrac{f\left( x \right) }{EI_{zs}\left( x \right) }dx}}, &{} x_{s\_st}<{ x_{Pm}}\leqslant x_{s\_end}\\ \displaystyle { \int _{x_A}^{x_B}{\dfrac{f\left( x_t \right) }{EI_{zt}\left( x_t \right) }dx_t}\!+\!\int _{x_B}^{x_{s\_st}}{\dfrac{f\left( x \right) }{EI_z\left( x \right) }dx}\!+\!\int _{x_{s\_st}}^{x_{s\_end}}{\dfrac{f\left( x \right) }{EI_{zs}\left( x \right) }dx}}\\ \displaystyle { \!+\!\int _{x_{s\_end}}^{x_{P_m}}{\dfrac{f\left( x \right) }{EI_z\left( x \right) }dx}}.&{} x_{s\_end}<{ x_{Pm}}\\ \end{array}\right. } \end{aligned}$$

4 $$\begin{aligned} \frac{1}{k_{s,i}}\!= & {} \!{\left\{ \begin{array}{ll} \displaystyle { \int _{x_A}^{x_B}{\dfrac{1.2\cos ^2\alpha _{P_m}}{GA_t\left( x_t \right) }dx_t}\!+\!\int _{x_B}^{x_{P_m}}{\dfrac{1.2\cos ^2\alpha _{P_m}}{GA\left( x \right) }dx}},&{} x_B<{ x_{Pm}}\leqslant x_{s\_st}\\ \displaystyle { \int _{x_A}^{x_B}{\dfrac{1.2\cos ^2\alpha _{P_m}}{GA_t\left( x_t \right) }dx_t}\!+\!\int _{x_B}^{x_{s\_st}}{\dfrac{1.2\cos ^2\alpha _{P_m}}{GA\left( x \right) }dx}}\\ \displaystyle { \!+\!\int _{x_{s\_st}}^{x_{P_m}}{\dfrac{1.2\cos ^2\alpha _{P_m}}{GA_s\left( x \right) }dx}},&{} x_{s\_st}<{ x_{Pm}}\leqslant x_{s\_end}\\ \displaystyle { \int _{x_A}^{x_B}{\dfrac{1.2\cos ^2\alpha _{P_m}}{GA_t\left( x_t \right) }dx_t}\!+\!\int _{x_B}^{x_{s\_st}}{\dfrac{1.2\cos ^2\alpha _{P_m}}{GA\left( x \right) }dx}}\\ \displaystyle { \!+\!\int _{x_{s\_st}}^{x_{s\_end}}{\dfrac{1.2\cos ^2\alpha _{P_m}}{GA_s\left( x \right) }dx}\!+\!\int _{x_{s\_end}}^{x_{P_m}}{\dfrac{1.2\cos ^2\alpha _{P_m}}{GA\left( x \right) }dx}}.&{} x_{s\_end}<{ x_{Pm}}\\ \end{array}\right. } \end{aligned}$$

In these equations, $\alpha _{P_m}$ is the operation angle. $x_A$ and $x_B$ denote the coordinates of the transaction curve starting point and ending point. $x_{s\_st}$ and $x_{x\_end}$ are the starting and ending coordinates of the tooth spall. *E* and *G* are Young’s modulus and shear modulus. $f\left( x \right) \!=\!\left[ \cos \alpha _{P_m}\left( x_{P_m}\!-\!x \right) \!-\!y_{P_m}\sin \alpha _{P_m} \right] ^2$, in which $x_{P_m}$ and $y_{P_m}$ are the coordinates of the mesh point $P_m$ in the *x* and *y* axis, respectively. $A_t(x_t)$, $I_{zt}(x_t)$, *A*(*x*), $I_z(x)$ denote the cross-sectional area and the area moment of inertia of transitional and healthy parts, respectively. $A_s(x)$, $I_{zs}(x)$ denote those of spalled part and are related to shape of the spalled defects.

For $k_h$, it is affected by the effective contact length in the presence of a spalling defect. In view of the nonlinear characteristics of the meshing process, an empirical formula is adopted in this paper as follows^28^:

5 $$\begin{aligned} k_{h,i}=\frac{{E^*}^{0.9}L_{e}^{0.8}F_i^{0.1}}{1.275}, \end{aligned}$$

where $E^*=\frac{2E_1E_2}{E_1+E_2}$, $L_e$ represents the effective contact length, $F_i$ is the meshing force of the *i*th tooth pair, $F_i=F\cdot lsr_i$, in which $lsr_i$ is the load-sharing ratio.

For $k_f$, it is caused by fillet-foundation deflection, and can be expressed as:

6 $$\begin{aligned} \frac{1}{k_{f,i}}=\frac{\cos ^2\alpha _{pm}}{ELC_{f}^{*}}\left\{ L^*\left( \frac{u_f}{S_f} \right) ^2+M^*\left( \frac{u_f}{S_f} \right) +P^*\left( 1+Q^*\tan ^2\alpha _{pm} \right) \right\} , \end{aligned}$$

where $u_f$, $S_f$, $L^*$, $M^*$, $P^*$ and $Q^*$ are constant parameters given in Ref.^29^, and $C_f^*$ is a coefficient utilized to correct the foundation stiffness within the double-tooth pair mesh region. The theoretical value of $C_f^*$ in the double tooth pair contact zone can be determined based on the model proposed in Refs.^16,30^ from ${{1}/{C_{f}^{*}={{k_{fA}}/{k_{fB}}}}}$, where $k_{fA}$ and $k_{fB}$ are the foundation stiffness of double and single tooth pairs at the transaction zone, see Ref.^30^. In this work, $C_{f}^{*}\approx 0.6$ in the double tooth pair contact area and $C_{f}^{*}=1$ in the single tooth pair mesh area.

### Asymmetric effect analysis and torsional stiffness

The basic mesh stiffness model has provided a general form of TVMS with spalling defects. However, the basic model has an implicit assumption that the center of spalling is located at the original point of *z*-axis. As the asymmetric spalling is typical in real operation, we tend to further discover and quantify this effect in this section.

**Figure 3:**
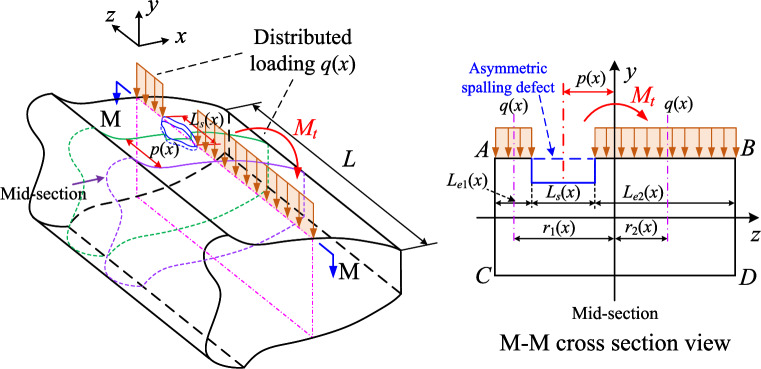
Introduction of torsional stiffness induced by asymmetric spalling defects.

An illustration of an asymmetric spalling defect is shown in Fig. 3, where the spalling defect deviates from the mid-section of the gear. *q*(*x*) is the distributed load of the meshing force *F* along the effective contact length $L_e(x)$, which is generally not significantly variable along the contact line. To facilitate the quick calculation of TVMS, we assume that *q*(*x*) is uniformly distributed along the contact line, i.e. $q(x)=F/L_e(x)$. The existence of asymmetric spalling defects results in an uneven load on both sides of the mid-section of the gear. The uneven load generates an additional torque $M_t$ around the *x*-axis, and thus generating a new form of stiffness. In this paper, we define it as the torsional stiffness, denoted as $k_t$.

According to the elastic mechanics and the beam theory, the torsional energy $U_t$ and the torsional stiffness $k_t$ can be obtained by the following expression:

7 $$\begin{aligned} {U_t=\dfrac{F^2}{2k_t}=\int _{x_{A}}^{x_B}{\frac{M_{t}^{2}\left( x_{Pm} \right) }{2GI_{pt}\left( x_t \right) }}dx_t+\int _{x_B}^{x_{s\_st}}{\frac{M_{t}^{2}\left( x_{Pm} \right) }{2GI_{p}\left( x \right) }}dx+\int _{x_{s\_st}}^{x_{Pm}}{\frac{M_{t}^{2}\left( x_{Pm} \right) }{2GI_{ps}\left( x \right) }}dx,} \end{aligned}$$

where $I_{pt}\left( x \right)$, $I_{p}\left( x \right)$, $I_{ps}\left( x \right)$ stands for the polar moments of inertia of the cross-sections on the transitional, healthy and spalled parts, respectively. Note that the polar moment of inertia $I_{ps}\left( x \right)$ is related to the offset of a spalling defect. Different defect position may result in different $I_{ps}\left( x \right)$, thus generating different torsional stiffness. For further details, please refer to “Equivalence of asymmetric spalling defects” section.

It can be further found from the cross-section view in Fig. 3 that the torque $M_t$ is related to the geometric feature of a spalling defect and can be calculated as:

8 $$\begin{aligned} M_t\left( x \right) =q\left( x \right) \left[ L_{e2}\left( x \right) r_2\left( x \right) -L_{e1}\left( x \right) r_1\left( x \right) \right] , \end{aligned}$$

where $L_{e1}$ and $L_{e2}$ are the effective contact lengths on both sides of a single spalling defect, $r_1$ and $r_2$ are the deviations of the center of the contact line from the mid-face in the *x*-axis cross-section of the spalled region, respectively.

Based on the geometric relationships, these features can be further written as:

9 $$\begin{aligned} \left\{ \begin{array}{c} L_{e1}\left( x \right) =\dfrac{1}{2}\left[ L-L_s\left( x \right) -2p\left( x \right) \right] \\ L_{e2}\left( x \right) =\dfrac{1}{2}\left[ L-L_s\left( x \right) +2p\left( x \right) \right] \\ r_1\left( x \right) =\dfrac{1}{4}\left[ L+L_s\left( x \right) +2p\left( x \right) \right] \\ r_2\left( x \right) =\dfrac{1}{4}\left[ L+L_s\left( x \right) -2p\left( x \right) \right] ,\\ \end{array} \right. \end{aligned}$$

where $p\left( x \right)$ is the offset of the central axis of the spalling defect from the central axis of the gear tooth. It should be noted that the offset $p\left( x \right)$ is not the same for different spalling shapes. For instance, for rectangular or elliptical shapes, the offset $p\left( x \right)$ is constant and does not change with *x*. In contrast, for irregular shapes, $p\left( x \right)$ can be determined by fitting the offset data at different *x* coordinates. Further details can be found in “Results and discussion” section.

Therefore, by combining Eqs. (8) and (9) into Eq. (7), the torsional stiffness can be further described as:

10 $$\begin{aligned} {\dfrac{1}{k_t}=\int _{x_A}^{x_B}{\dfrac{L_{s}^{2}\left( x_{Pm} \right) p^2\left( x_{Pm} \right) }{L_{e}^{2}\left( x_{Pm} \right) GI_{pt}\left( x_t \right) }}dx_t+\int _{x_B}^{x_{s\_st}}{\dfrac{L_{s}^{2}\left( x_{Pm} \right) p^2\left( x_{Pm} \right) }{L_{e}^{2}\left( x_{Pm} \right) GI_{p}\left( x \right) }}dx+\int _{x_{s\_st}}^{x_{Pm}}{\dfrac{L_{s}^{2}\left( x_{Pm} \right) p^2\left( x_{Pm} \right) }{L_{e}^{2}\left( x_{Pm} \right) GI_{ps}\left( x \right) }}dx.} \end{aligned}$$

It is apparent that the torque is present in cases of asymmetric spalling, where $p(x)\ne 0$. Consequently, torsional stiffness $k_t$ is limited to the region of asymmetric spalling. According to the above discussion, the total stiffness of the gear system with asymmetric spalling defects can be modified by taking into account $k_t$, given as follows:

11 $$\begin{aligned} \frac{1}{k_{total}}\!=\!\sum _{i=1}^n{\left( \frac{1}{k_{ag,i}}\!+\!\frac{1}{k_{bg,i}}\!+\!\frac{1}{k_{sg,i}}\!+\!\frac{1}{k_{tg,i}}\!+\!\frac{1}{k_{fg,i}}\!+\!\frac{1}{k_{ap,i}}\!+\!\frac{1}{k_{bp,i}}\!+\!\frac{1}{k_{sp,i}}\!+\!\frac{1}{k_{tp,i}}\!+\!\frac{1}{k_{fp,i}}\!+\!\frac{1}{k_{h,i}} \right) }. \end{aligned}$$

## Shape-independent TVMS evaluation with asymmetric spalling defects

### Equivalence of asymmetric spalling defects

According to the TVMS model presented in the above section, the key point to evaluate the TVMS of a gear with asymmetric spalling is to capture the cross-sectional properties ($A_s$, $I_{zs}$, $I_{ps}$) and the effective contact length $L_e$ within the spalling area. Due to the different shape and random distribution of spalling defects, it is usually inaccurate and difficult to directly describe the effect of tooth spalling using simple geometries (rectangular, circular, etc.) as approximations. By adopting the basic ideas of shape equivalence given by Luo et al.^25^ , we hereby propose a shape-independent method for asymmetric spalling defects.

**Figure 4:**
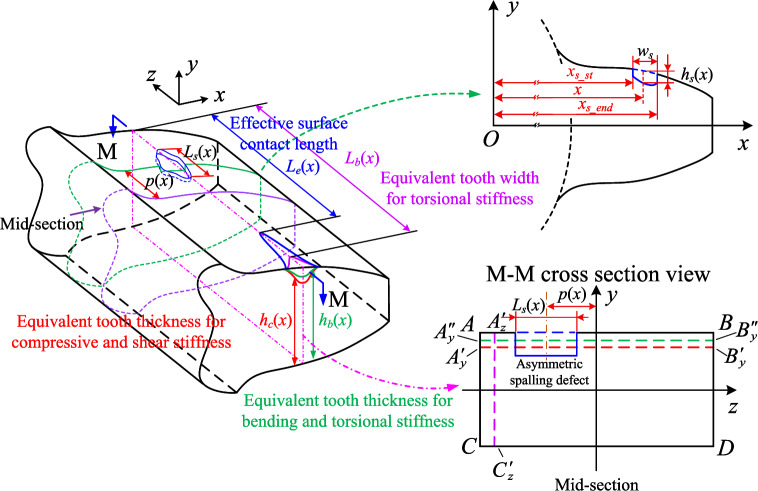
Equivalence of asymmetric spalling defects.

The essence of the shape-independent method is to construct equivalent rectangles to characterize the effect of asymmetric spalling defects on the cross-sectional properties. There are two cases for the cross-section properties ($A_s$, $I_{zs}$, $I_{ps}$).

(1) Cross-sectional area $A_s$ and moment of inertia of *z*-axis $I_{zs}$

First, we assume that the spalling depth $h_s(x)$ is constant along the *z*-axis at any *x*-coordinate within the spalling region. This means that the spalled cross-section is rectangular. As shown in Fig. 4, two rectangles $A_{y}^{'}B_{y}^{'}DC$ and $A_{y}^{''}B_{y}^{''}DC$ are used to be equivalent to the actual area $A_s(x)$ and the moment of inertia of *z*-axis $I_{zs}(x)$ of the spalled cross-section in *x*-coordinate, i.e. the following conditions are satisfied^25^:

12 $$\begin{aligned} {\left\{ \begin{array}{ll} A_s\left( x \right) =S_{A_{y}^{'}B_{y}^{'}CD}=Lh_c\left( x \right) =2Lh\left( x \right) -L_s\left( x \right) h_s\left( x \right) \\ I_{zs}\left( x \right) =I_{z,A_{y}^{''}B_{y}^{''}CD}=\left( 1/12 \right) h_{b}^{3}\left( x \right) L\\ =\dfrac{2Lh\left( x \right) ^3}{3}\!+\!2Lh\left( x \right) \delta _{ys}^{2}\left( x \right) \!-\!\left[ \dfrac{L_s\left( x \right) h_{s}^{3}\left( x \right) }{12}\!+\!L_s\left( x \right) h_s\left( x \right) \left( h\left( x \right) \!-\!\dfrac{1}{2}h_s\left( x \right) \!+\!\delta _{ys}\left( x \right) \right) ^2 \right] ,\\ \end{array}\right. } \end{aligned}$$

where $h_c(x)$ and $h_b(x)$ is the heights of the rectangle $A_{y}^{'}B_{y}^{'}DC$ and rectangle $A_{y}^{''}B_{y}^{''}DC$. $\delta _{ys}\left( x \right) ={{\left[ \left( h\left( x \right) -{{h_s}/{2}} \right) L_sh_s \right] }/{A_s\left( x \right) }}$, $L_s(x)$ is the length of the spalling defect, *h*(*x*) is the half tooth thickness on the involute curve.

Under these conditions, the compression and shear stiffnesses under various spalling shapes can be determined based on the area of the equivalent rectangle $A_{y}^{'}B_{y}^{'}DC$. Similarly, the bending stiffness can be found by calculating the moment of inertia of the equivalent rectangle $A_{y}^{''}B_{y}^{''}DC$. It is possible to define $h_c(x)$ as the equivalent thickness for axial compression stiffness and shear stiffness, whereas $h_b(x)$ represents the equivalent thickness of bending stiffness. Hence, the aim of this equivalence is to establish the correlation between the two forms of equivalent thicknesses and the spalling shape parameters. We adopt the two defect ratios defined by Luo et al.^25^ to quantify the degree of spalling, which can be expressed as:

13 $$\begin{aligned} {\left\{ \begin{array}{ll} C_{Ls}\left( x \right) = \dfrac{L_s\left( x \right) }{L}\\ C_{hs}\left( x \right) = \dfrac{h_s\left( x \right) }{ 2h\left( x \right) },\\ \end{array}\right. } \end{aligned}$$

where $C_{Ls}(x)$, $C_{hs}(x)$ are the defect length ratio and defect depth ratio of the tooth spalling at displacement *x*, respectively.

Combining the cross-sectional area under spalling as well as the moment of inertia (see Eq. (12)), the form of these two equivalent thicknesses have been derived as

14 $$\begin{aligned} {\left\{ \begin{array}{ll} h_b\left( x \right) = 2h\left( x \right) \left[ K_c\left( x \right) \right] ^{\frac{1}{3}}\\ h_c\left( x \right) = 2\left[ 1-C_{Ls}\left( x \right) C_{hs}\left( x \right) \right] h\left( x \right) , \\ \end{array}\right. } \end{aligned}$$

with

15 $$\begin{aligned} K_c\left( x \right) =\frac{1+C_{hs}^{4}\left( x \right) C_{Ls}^{2}\left( x \right) -\left[ 4C_{hs}^{3}\left( x \right) -6C_{hs}^{2}\left( x \right) +4C_{hs}\left( x \right) \right] C_{Ls}\left( x \right) }{1-C_{hs}\left( x \right) C_{Ls}\left( x \right) }. \end{aligned}$$

(2) The polar moment of inertia $I_{ps}$

As discussed in previous section, the effect of asymmetric spalling defects is mainly manifested through the torsional stiffness. Therefore, the focal point of TVMS evaluation is computing the polar moment of inertia under asymmetric spalling defects. The polar moment of inertia $I_{ps}$ is the square moment of the area with respect to the pole, which can be expressed by the transformation $I_{ps}=I_{ys}+I_{zs}$, where $I_{ys}$ and $I_{zs}$ denote the moment of inertia of the *y* axis and *z* axis, respectively. Since $I_{zs}$ has been derived through the rectangle $A_{y}^{''}B_{y}^{''}DC$, we hereby mainly focus on the equivalence of $I_{ys}$. Specifically, since the reduction of the cross-sectional area causes a change in the central axis of the moment of inertia of an area in the *z*-axis, the rectangle $A_{z}^{'}B_{z}^{'}DC$ is introduced to be equivalent to $I_{ys}$, as shown in Fig. 4, and the following condition satisfies:

16 $$\begin{aligned} I_{ys}\left( x \right) =I_{y,A_{z}^{'}B_{z}^{'}DC}=\dfrac{1}{6} hL_{b}\left( x \right) ^{3}, \end{aligned}$$

where $L_b(x)$ is the equivalent tooth width for torsional stiffness.

**Figure 5:**
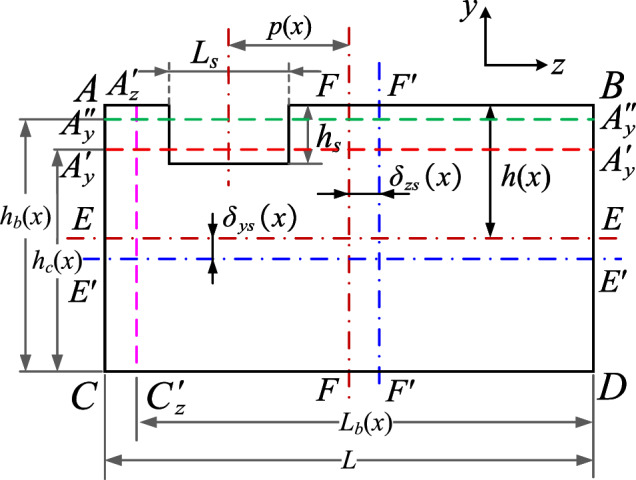
Cross-section model of the tooth with asymmetric spalling.

Based on the above equivalence, a key point is how the equivalent width $L_b(x)$ is calculated. For asymmetric spalling of gear tooth, as depicted in Figs. 4 and 5, the change $\delta _{zs}$ in the central axis of the moment of inertia of an area in the *z*-axis can be expressed as:

17 $$\begin{aligned} \delta _{zs}\left( x \right) =\dfrac{p\left( x \right) L_s\left( x \right) h_s\left( x \right) }{A_s\left( x \right) }. \end{aligned}$$

According to parallel axis theorem, the moment of inertia of *y*-axis $I_{ys}$ with respect to the changed central axis $F^{'}F^{'}$ can be evaluated by

18 $$\begin{aligned} I_{ys}\left( x \right) =\dfrac{h\left( x \right) L^3}{6}+2Lh\left( x \right) \delta _{zs}^{2}\left( x \right) -\left[ \dfrac{h_s\left( x \right) L_{s}^{3}\left( x \right) }{12}+L_s\left( x \right) h_s\left( x \right) \left( p\left( x \right) +\delta _{zs}\left( x \right) \right) ^2 \right] . \end{aligned}$$

Additionally, we further define the defect offset ratio $C_{ps}$ to reflect the effect of spalling defect location, i.e:

19 $$\begin{aligned} C_{ps}\left( x \right) = \dfrac{p\left( x \right) }{L}. \end{aligned}$$

By substituting Eq. (19) into Eq. (18) and Eq. (16), the equivalent width $L_b(x)$ can be obtained as:

20 $$\begin{aligned} L_b\left( x \right) =L\left[ K_p\left( x \right) \right] ^{\frac{1}{3}}, \end{aligned}$$

with

21 $$\begin{aligned} K_p\left( x \right) =\frac{1+C_{hs}^{2}\left( x \right) C_{Ls}^{4}\left( x \right) -C_{hs}\left( x \right) C_{Ls}^{3}\left( x \right) -\left[ 1+12C_{ps}^{2}\left( x \right) \right] C_{hs}\left( x \right) C_{Ls}\left( x \right) }{1-C_{hs}\left( x \right) C_{Ls}\left( x \right) }. \end{aligned}$$

Combining the effects of the spalling defect on the area moments of inertia $I_{ys}\left( x \right)$ and $I_{zs}\left( x \right)$, the polar moment of inertia $I_{ps}\left( x \right)$ can be written as:

22 $$\begin{aligned} I_{ps}\left( x \right) =I_{ys}\left( x \right) +I_{zs}\left( x \right) =\dfrac{1}{6}K_p\left( x \right) h\left( x \right) L^3+\dfrac{2}{3}K_c\left( x \right) h^3\left( x \right) L. \end{aligned}$$

According to the above discussion, the equivalent thickness curves ($h_c(x)$ and $h_b(x)$) and equivalent length curve $L_b(x)$ within the spalling region can be determined directly after specifying the defect rations ($C_{Ls}$, $C_{hs}$ and $C_{ps}$). We hereby summarize the cross-sectional properties as:

23 $$\begin{aligned} \left\{ \begin{aligned} A_s\left( x \right)&=Lh_c\left( x \right) =2\left[ 1-C_{Ls}\left( x \right) C_{hs}\left( x \right) \right] Lh\left( x \right) \\ I_{zs}\left( x \right)&=\frac{2}{3}K_c\left( x \right) Lh^3\left( x \right) \\ I_{ps}\left( x \right)&=I_{ys}\left( x \right) +I_{zs}\left( x \right) =\frac{1}{6}K_p\left( x \right) h\left( x \right) L^3+\frac{2}{3}K_c\left( x \right) h^3\left( x \right) L.\\ \end{aligned} \right. \end{aligned}$$

Since $C_{Ls}(x)$, $C_{hs}(x)$ and $C_{ps}(x)$ are all modeled as functions of *x*, the Eq. (23) is able to quantify the effect of spalling defects with different shapes and different locations. Further discussion is provided below for these particular types of features, respectively.

### TVMS formula for gears with asymmetric spalling defects

As mentioned above, the morphology and location of the spalling defect were quantified by the established defect ratios (Eqs. (13) and (19)) and further transformed into cross-sectional characteristic parameters of the equivalence rectangles. As a result, by inserting Eq. (23) into Eqs. (2)–(4) and (10), the axial compressive, bending, shear and torisonal stiffnesses for a gear with asymmetric spalling defect can be written as a unified equation as follows:

24 $$\begin{aligned} \frac{1}{k_{a,i}}\!= & {} \!{\left\{ \begin{array}{ll} \displaystyle { \int _{x_A}^{x_B}{\dfrac{\sin ^2\alpha _{Pm}}{EA_{t}(x_t)}}dx_t\!+\!\int _{x_B}^{x_{Pm}}{\dfrac{\sin ^2\alpha _{Pm}}{EA(x)}}dx},&{} x_B<{ x_{Pm}}\leqslant x_{s\_st}\\ \displaystyle { \int _{x_A}^{x_B}{\dfrac{\sin ^2\alpha _{Pm}}{EA_{t}(x_t)}}dx_t\!+\!\int _{x_B}^{x_{s\_st}}{\dfrac{\sin ^2\alpha _{Pm}}{EA(x)}}dx}\\ +\displaystyle { \int _{x_{s\_st}}^{x_{Pm}}{\dfrac{\sin ^2\alpha _{Pm}}{2EL\left( 1-C_{Ls}\left( x \right) C_{hs}\left( x \right) \right) h\left( x \right) }}dx},&{} x_{s\_st}<{ x_{Pm}}\leqslant x_{s\_end}\\ \displaystyle { \int _{x_A}^{x_B}{\dfrac{\sin ^2\alpha _{Pm}}{EA_{t}(x_t)}}dx_t\!+\!\int _{x_B}^{x_{s\_st}}{\dfrac{\sin ^2\alpha _{Pm}}{EA(x)}}dx}\\ \displaystyle { \!+\!\int _{x_{s\_st}}^{x_{s\_end}}{\dfrac{\sin ^2\alpha _{Pm}}{2EL\left( 1-C_{Ls}\left( x \right) C_{hs}\left( x \right) \right) h\left( x \right) }}dx\!+\!\int _{x_{s\_end}}^{x_{Pm}}{\dfrac{\sin ^2\alpha _{Pm}}{EA(x)}}dx}.&{} x_{s\_end}<{ x_{Pm}}\\ \end{array}\right. } \end{aligned}$$

25 $$\begin{aligned} \frac{1}{k_{b,i}}= & {} {\left\{ \begin{array}{ll} \displaystyle { \int _{x_A}^{x_B}{\dfrac{f\left( x_t \right) }{EI_{zt}\left( x_t \right) }}dx_t+\int _{x_B}^{x_{Pm}}{\dfrac{f\left( x \right) }{EI_z\left( x \right) }dx}},&{} x_B<{ x_{Pm}}\leqslant x_{s\_st}\\ \displaystyle { \int _{x_A}^{x_B}{\dfrac{f\left( x_t \right) }{EI_{zt}\left( x_t \right) }dx_t}+\int _{x_B}^{x_{s\_st}}{\dfrac{f\left( x \right) }{EI_z\left( x \right) }dx}}\\ \displaystyle { +\int _{x_{s\_st}}^{x_{Pm}}{\dfrac{f\left( x \right) }{\left( 2/3 \right) K_c\left( x \right) Lh^3\left( x \right) }dx}},&{} x_{s\_st}<{ x_{Pm}}\leqslant x_{s\_end}\\ \displaystyle { \int _{x_A}^{x_B}{\dfrac{f\left( x_t \right) }{EI_{zt}\left( x_t \right) }dx_t}+\int _{x_B}^{x_{s\_st}}{\dfrac{f\left( x \right) }{EI_z\left( x \right) }dx}}\\ \displaystyle { +\int _{x_{s\_st}}^{x_{s\_end}}{\dfrac{f\left( x \right) }{\left( 2/3 \right) K_c\left( x \right) Lh^3\left( x \right) }dx}+\int _{x_{s\_end}}^{x_{Pm}}{\dfrac{f\left( x \right) }{EI_z\left( x \right) }dx}}.&{} x_{s\_end}<{ x_{Pm}}\\ \end{array}\right. } \end{aligned}$$

26 $$\begin{aligned} \frac{1}{k_{s,i}}= & {} {\left\{ \begin{array}{ll} \displaystyle { \int _{x_A}^{x_B}{\dfrac{1.2\cos ^2\alpha _{Pm}}{GA_t\left( x_t \right) }dx_t}+\int _{x_B}^{x_{Pm}}{\dfrac{1.2\cos ^2\alpha _{Pm}}{GA\left( x \right) }dx}},&{} x_B<{ x_{Pm}}\leqslant x_{s\_st}\\ \displaystyle { \int _{x_A}^{x_B}{\dfrac{1.2\cos ^2\alpha _{Pm}}{GA_t\left( x_t \right) }dx_t}+\int _{x_B}^{x_{s\_st}}{\dfrac{1.2\cos ^2\alpha _{Pm}}{GA\left( x \right) }dx}}\\ \displaystyle { +\int _{x_{s\_st}}^{x_{Pm}}{\dfrac{1.2\cos ^2\alpha _{Pm}}{2G\left[ 1-C_{Ls}\left( x \right) C_{hs}\left( x \right) \right] Lh\left( x \right) }dx}},&{} x_{s\_st}<{ x_{Pm}}\leqslant x_{s\_end}\\ \displaystyle { \int _{x_A}^{x_B}{\dfrac{1.2\cos ^2\alpha _{Pm}}{GA_t\left( x_t \right) }dx_t}+\int _{x_B}^{x_{s\_st}}{\dfrac{1.2\cos ^2\alpha _{Pm}}{GA\left( x \right) }dx}}\\ \displaystyle { \!+\!\int _{x_{s\_st}}^{x_{s\_end}}{\dfrac{1.2\cos ^2\alpha _{Pm}}{2G\left[ 1-C_{Ls}\left( x \right) C_{hs}\left( x \right) \right] Lh\left( x \right) }dx}\!+\!\int _{x_{s\_end}}^{x_{Pm}}{\dfrac{1.2\cos ^2\alpha _{Pm}}{GA\left( x \right) }dx}}.&{} x_{s\_end}<{ x_{Pm}}\\ \end{array}\right. } \end{aligned}$$

27 $$\begin{aligned} \frac{1}{k_{t,i}}= & {} {\left\{ \begin{array}{ll} 0,&{} x_B<{ x_{Pm}}\leqslant x_{s\_st}\\ {\displaystyle {\int _{x_A}^{x_B}{\dfrac{L_{s}^{2}\left( x_{Pm} \right) p^2\left( x_{Pm} \right) }{\left[ L-L_s\left( x_{Pm} \right) \right] ^2GI_{pt}\left( x_t \right) }dx_t}\!+\!\int _{x_B}^{x_{s\_st}}{\dfrac{L_{s}^{2}\left( x_{Pm} \right) p^2\left( x_{Pm} \right) }{\left[ L-L_s\left( x_{Pm} \right) \right] ^2GI_p\left( x \right) }dx}}}\\ {\displaystyle {+\int _{x_{s\_st}}^{x_{Pm}}{\dfrac{L_{s}^{2}\left( x_{Pm} \right) p^2\left( x_{Pm} \right) }{\left[ L\!-\!L_s\left( x_{Pm} \right) \right] ^2G\left[ \dfrac{1}{6}K_p\left( x \right) h\left( x \right) L^3\!+\!\dfrac{2}{3}K_c\left( x \right) h^3\left( x \right) L \right] }dx}},}&{} x_{s\_st}<{ x_{Pm}}\leqslant x_{s\_end}\\ 0.&{} x_{s\_end}<{ x_{Pm}}\\ \end{array}\right. } \end{aligned}$$

**Figure 6:**
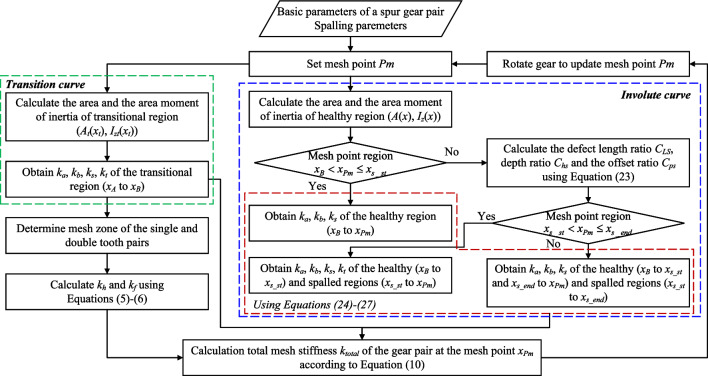
Flowchart of the mesh stiffness calculation process for a spur gear pair with asymmetric spalling defects.

The Hertzian contact stiffness and the fillet-foundation stiffness are not affected by the offset of the spalling defect and can still be expressed in terms of Eqs. (5) and (6). Finally, the total stiffness during the meshing cycle can be calculated using Eq. (11). The calcuation process of the mesh stiffness is illustrated in Fig. 6. Note that the proposed method provides a general calculation model for various types of spalling defects, which is more convenient for TVMS evaluation.

## Simulation validation method for asymmetric spalling

**Figure 7:**
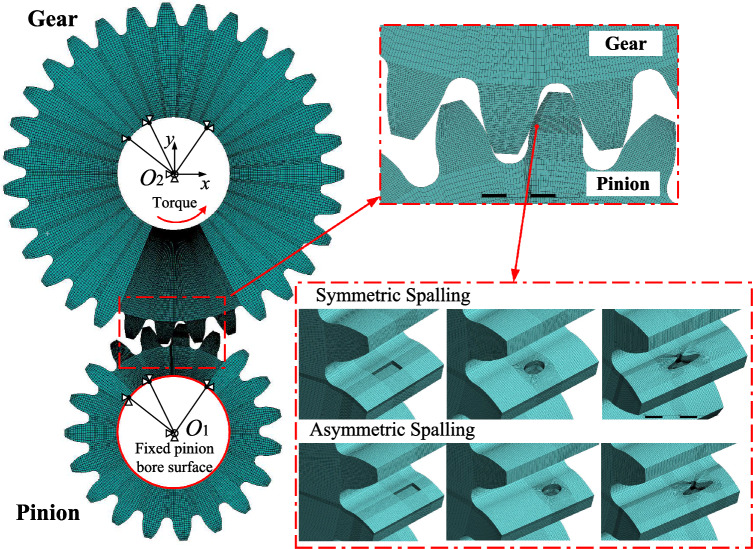
A finite element model of a spur gear pair with different spalling shapes under symmetric and asymmetric conditions (rectangular, elliptical and irregular shapes).

In order to validate the proposed asymmetric mesh stiffness model derived in this paper, typical simple shapes (rectangular, elliptical) as well as irregular shapes of spalling defect at symmetric and asymmetric locations for the same mesh pair is evaluated in this section using the finite element method (FEM) for comparison, as shown in Fig. 7. The pinion is modeled as a faulty gear with a spalled tooth, and the gear is modeled as a healthy gear with no tooth fault. The parameters of the spur gear pair are listed in Table 1. In order to improve the calculation accuracy and efficiency, the element shape of the gear body and gear teeth is mapped hexahedral using HyperMesh software. The gear and pinion are both uniformly meshed with 60 layers in the direction of the teeth width, with the mesh locally encrypted around the spalling region.

**Table 1:** Parameters of the gear-pinion set.

Parameters	Value	Parameters	Value
Number of pinon teeth	19	Module *m* (*mm*)	3.2
Number of gear teeth	31	Young’s modulus *E* (GPa)	206.8
Teeth width *L* (*mm*)	21.5	Possion’s ratio $\nu$	0.3
Pressure angle $\alpha$ ($\circ$)	20	Addendum coefficient $h_a$	1
Hube bore radius (*mm*)	17.5	Tip clearance coefficient *c*	0.25

At present, there are two main types of methods for solving the mesh stiffness by FEM: one is to calculate the rectilinear stiffness directly from the relationship between contact force *F* and deflection along the action line $\delta$, i.e. $k=F/\delta$^31^. The other method is to evaluate gear mesh stiffness using $k={{T}/{\theta _t r_{b}^{2}}}$ with angular deflection as the medium, where $\theta _t$ is the angular displacement of the gear bore^8,32^. The latter avoids the need for repeated measurement extraction of rectilinear deflection for each mesh point, which is easier to apply. In this respect, this paper adopts the basic ideas of the second method.

It is significant to note that this method has good applicability to healthy gear pair by selecting the minimum angular deflection of an end surface circle of the gear bore. However, in the spalling region, the angular deflection distribution of the gear bore is not uniform along the axial direction because the meshing force acts on the remaining healthy contact surface, which is no longer uniformly distributed. Directly selecting the minimum angular deflection of one circle of the bore or averaging the minimum angles of several circles cannot reflect the true meshing stiffness characteristics. To address the above issues, this paper further improves the method through considering the loading state of each layer of the meshes along the axial direction and extracting the results of the mesh stiffness under the three-dimensional spalling defect.

The setting of the FEM is shown in Fig. 7, where all the degrees of freedom on the pinion bore surface are fully constrained. A cylindrical coordinate system is set up on the gear bore surface. The gear only has rotational degree of freedom. A torque ($T=100N\cdot m$) is applied on the bore surface of the gear in counterclockwise direction. The friction of surface contact is not considered. The tangential deflection $u_y$ of all nodes of the gear bore surface are extracted along the *y*-direction of the cylindrical coordinate system after using the static solution. The angular deflection $\theta _t$ on the bore is calculated to be $u_y/r_{bore}$, where $r_{bore}$ is the radius on the gear bore. In addition, all the nodes are sorted and categorised according to the value of the *z*-coordinate. Nodes with the identical *z*-coordinate are the same layer of circles parallel to the gear body end face and the smallest tangential deflection in each circle is extracted.

**Figure 8:**
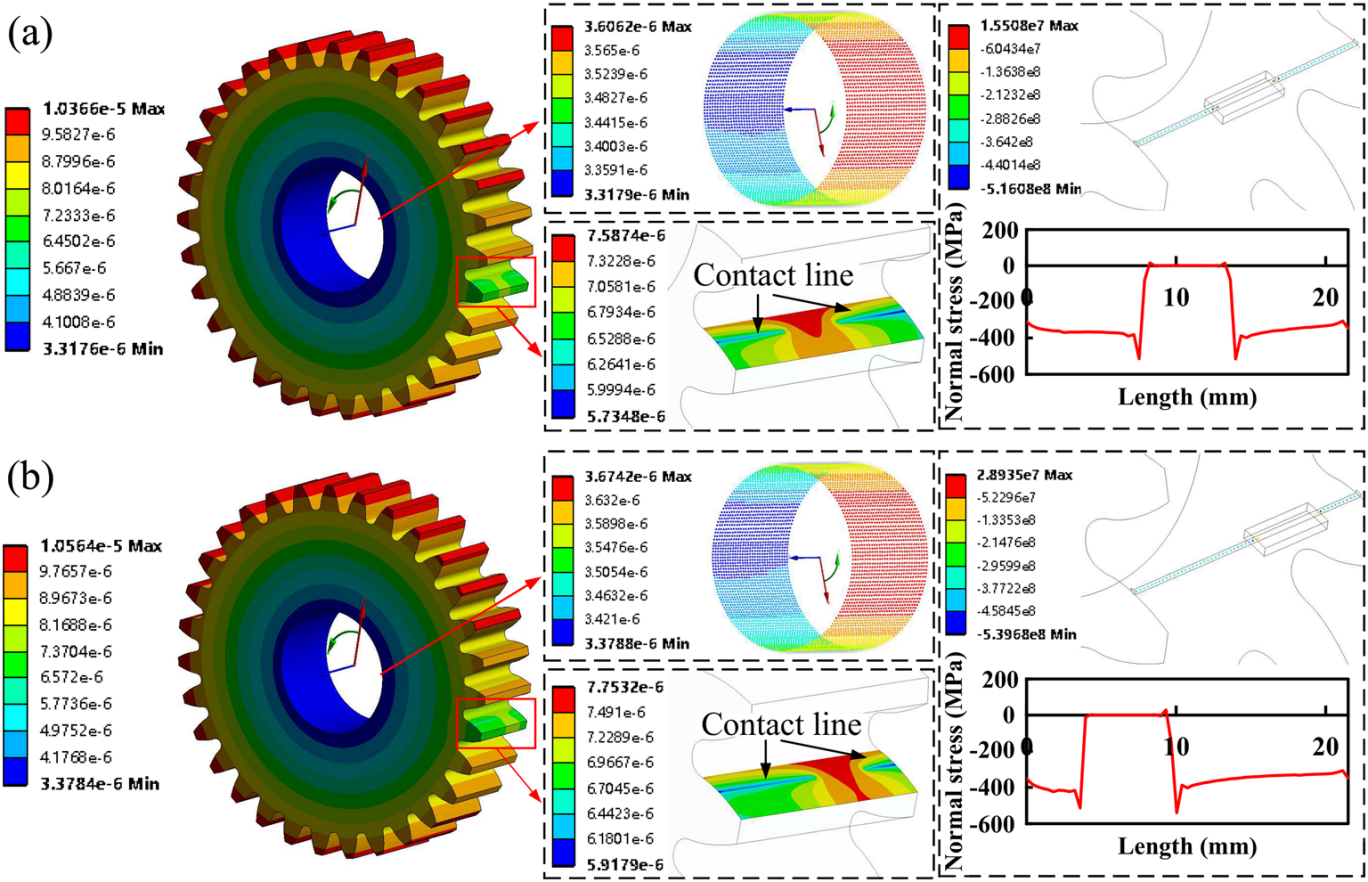
The distributions of tangential deflection and normal stress in the bore and contact tooth surfaces at symmetric and asymmetric locations.

According to the above setup, different layers are distinguished based on the meshing state, as illustrated in Fig. 8. It is evident that, in both symmetric and asymmetric conditions, the nodal normal stresses on the contact line remain largely unaltered, with the exception of the abrupt change in normal stresses at the nodes near the spalling defects due to stress concentration. This further substantiates the rationale behind the adoption of the uniform loading assumption for the distributed load *q*(*x*) in the proposed model. Moreover, the location of the spalling will affect the tangential deflection distribution of the meshing gear tooth as well as the tangential deflection distribution of bore surface. Essentially, it is the area of the tooth within the remaining effective contact line that is actually loaded. In this regrad, we classify the meshes into two categories based on whether the meshes lie on the contact line or not: supporting meshes and non-supporting meshes. The set of layers of the circle corresponding to the *z*-coordinate of the supporting mesh is denoted as *V* and the set corresponding to the non-supporting mesh is denoted as *S*. If the gear is sliced in terms of meshes along the axial direction, it can be seen that only the supporting meshes play a real role in the stiffness. Therefore, in this paper, the total stiffness of the gear is defined as the sum of the stiffnesses of all the slices of the gear tooth corresponding to the supporting mesh, i.e. $k_{total,FEM}=\sum _{k=1}^n{\sum _{i\in {V_k}}{k_{i,k}}}$, where $k_{i,k}$ is the stiffness of the gear tooth corresponding to the *i*th layer of the bore circle mesh for the *k*th meshing gear tooth.

Furthermore, by combining the extracted tangential deflection data of the bore nodes with the stiffness calculation method proposed in Ref.^8^, the mesh stiffness in the FEM method can be further expressed as:

28 $$\begin{aligned} k_{total,FEM}={\left\{ \begin{array}{ll} \dfrac{Tr_{bore}}{r_{b}^{2}\left( L-L_s \right) }\displaystyle \sum \limits _{j=1,i\in V}^{n_{circle,i}}{\dfrac{\Delta z_i}{\min \left( u_{y,ij} \right) }},&{} \mathrm {single-tooth-pair}\\ \dfrac{Tr_{bore}}{r_{b}^{2}\left( 2L-L_s \right) }\displaystyle \sum \limits _{k=1}^2{\sum _{j=1,i\in V_k}^{n_{circle,i}}{\dfrac{\Delta z_i}{\min \left( u_{y,ij} \right) }}},&{} \mathrm {double-tooth-pair}\\ \end{array}\right. } \end{aligned}$$

where $n_{circle,i}$ and $\Delta z_i$ are the number of nodes and the mesh size of the bore circle in the *i*th layer, respectively. $\min \left( u_{y,ij} \right)$ represents the minimum value of tangential deflection of all nodes of the bore circle of the *i*th layer.

In order to obtain the meshing stiffness under a complete mesh cycle, the contact point is set from the pinion tooth addendum circle to the end of the complete disengagement of this meshing gear tooth pair. During this process, the pinion is rotated counter-clockwise in $1^\circ$ increments and each different state is simulated statically in turn to extract the tangential displacement of the gear’s bore surface. Based on Eq. (28), MATLAB code was written to calculate the mesh stiffness for different types of spalling conditions during a mesh cycle.

## Results and discussion

The following subsections validate the effectiveness of the proposed model in dealing with asymmetric conditions under different spalling shapes.

### Case 1: rectangular shape

**Figure 9:**
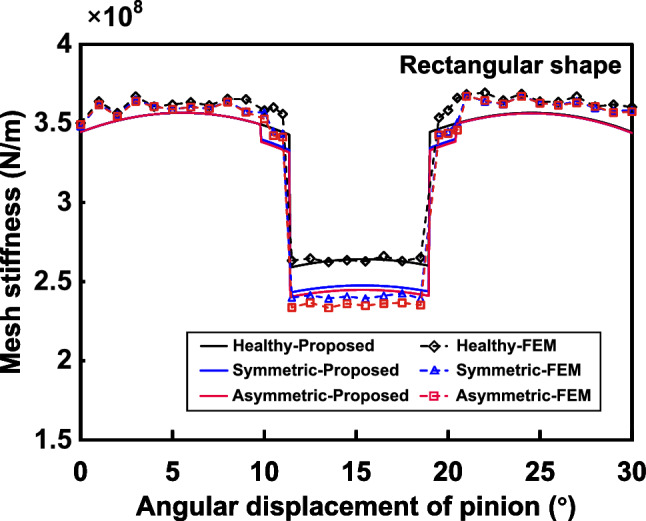
Comparison of the TVMS under symmetric and asymmetric rectangular spalling evaluated by proposed method and FEM.

**Figure 10:**
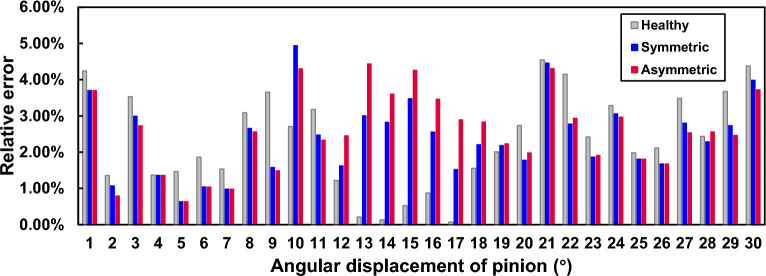
Relative errors between the proposed model and FEA calculations under symmetric and asymmetric rectangular spalling.

The parameters for the rectangular spall, are $L_s=6mm$, $w_s=1.8mm$, $h_s=0.6mm$, $x_{s\_st}=29.295mm$. In order to verify the asymmetric spalling effect, we set the offset *p*(*x*) of the spalling center to be 4*mm*, and compared it with symmetric rectangular spalling ($p(x)=0$). In addition, the TVMS of health gear is also extracted for proposed model and FEM as a benchmark. The TVMS results and the relative errors are shown in Figs. 9 and 10, respectively.

The trends and values of TVMS obtained from the model calculation and the simulation are basically in agreement, whenever the tooth is healthy or spalled. The maximum relative error is $4.44\%$ (pinion rotation $13^\circ$), which is in the acceptable range. The differences are mainly due to: a) the simulation result is affected by many uncertainties such as mesh size and type, etc, b) the contact line is located at the two end faces of the gear and the edge of the spalled region is prone to stress concentration, which makes the displacements near the mesh in this region have large sudden changes. In addition, since the inverse of torsional stiffness $1/{k_t}$ is equal to 0 under the symmetric condition, this TVMS model is identical to the one proposed in Ref.^25^. Consequently, a comparison of the computational results of the TVMS model under both symmetric and asymmetric conditions with the simulation results under asymmetric condition reveals that the maximum relative error of the model proposed in this paper ($4.44\%$) is smaller than that of the existing model based on the symmetric assumption ($5.63\%$). This further illustrates the effectiveness of the constructed model in describing asymmetric spalling.

Comparing the symmetric and asymmetric results, it is found that the offset of the spalling region center has caused a further decrease in the mesh stiffness. Since the length, width and height of the rectangular spalling are constant values, the amount of change in TVMS is essentially the same for symmetric and asymmetric positions during the rotation of the gear pair. Particularly, the TVMS reduction in the single-tooth meshing zone is greater than that in the double-tooth meshing zone. This is because the meshing force in single-tooth meshing is concentrated on the effective contact line of a single tooth, which makes the asymmetric torque $M_t$ larger than that in double-tooth meshing, and therefore the effect of torsional stiffness is more significant.

**Figure 11:**
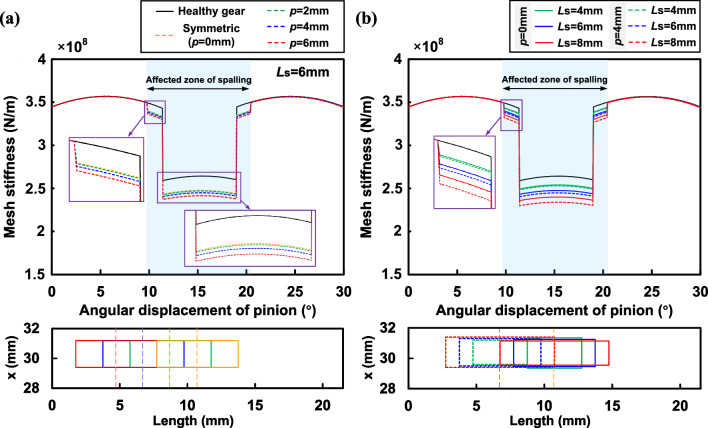
TVMS of proposed method for rectangular spalling with different offsets and spalling lengths.

**Figure 12:**
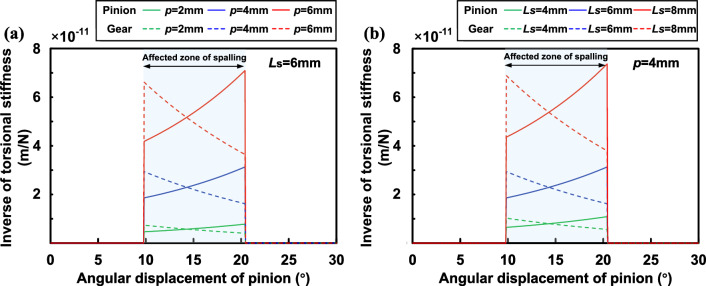
Inverse of torsional stiffness $1/k_t$ for rectangular spalling with different offsets and spalling lengths.

In addition, the effects of offset and spalling length on TVMS are further discussed. TVMS of gears with rectangular spalling under the offsets of 2*mm*, 4*mm* and 6*mm* are obtained and those of healthy and symmetric ($p=0mm$) spalling are also calculated for comparison as shown in Fig. 11a. It can be seen that as the offset increases there is a continuous reduction in the mesh stiffness. Furthermore, the farther away from the mid-face the spalling is, the greater the reduction in mesh stiffness. This phenomenon can be explained by the torsional stiffness model. In order to present the effect of torsional stiffness in a more intuitive manner, the inverse of the torsional stiffness $1/{k_t}$ at the aforementioned offsets is extracted further, as shown in Fig. 12a. It can be observed that the offset of the spalling changes the torsional stiffness by affecting the torque, and its effect is shown to be non-linear. The non-linear effect may keep the same as the pinion rotates in the affected zone.

As for the effect of spalling length, we have calculated TVMS for rectangular spalling with lengths of 4*mm*, 6*mm* and 8*mm* under symmetric ($p=0mm$) and asymmetric ($p=4mm$) conditions as shown in Fig. 11b. Furthermore, the torsional stiffness under asymmetric conditions is extracted and presented in Fig. 12b. The comparison shows that the effect of torsional stiffness on the total stiffness becomes more significant as the spalling length increases, even for the same offset. This also shows that the asymmetric effect is a problem that cannot be ignored when the spalling defect size and the offset are large.

### Case 2: elliptical/round shape

Ellipses and circles are the more common spalling shapes for gear pairs in actual operation. The corresponding finite element model is shown in case 2 of Fig. 7. This section focuses on the TVMS model for the elliptical shape since the circle can be considered as a special case of the ellipse. The key geometric features that determine the elliptical shape include maximum length $L_{s\_max}$, maximum width $w_{s\_max}$ and depth $h_s$. In this case, we set $L_{s\_max}=4mm$, $w_{s\_max}=2mm$ and the depth $h_s=1mm$. In addition, the position of the spalling depends on the coordinates $(x_{s\_center}, y_{s\_center})$ of the elliptic centre, wherein in this case $x_{s\_center}=30.295mm$ and $y_{s\_center}=L/2+p(x)$. For any *x*, we assume the spalling depth $h_s$ keeps constant.

Based on the geometric relationships for the ellipse, the spalling length varies with *x*, which can be expressed as:

29 $$\begin{aligned} L_s\left( x \right) =L_{s\_max}\sqrt{1-\frac{4\left( x-x_{s\_center} \right) ^2}{w_{s\_max}^{2}}}, x\in \left[ x_{s\_st},x_{s\_end} \right] \end{aligned}$$

where $x_{s\_st}=x_{s\_center}-w_{s\_max}/2$, $x_{s\_end}=x_{s\_center}+w_{s\_max}/2$ are the starting and ending position of the elliptical spalling, respectively.

**Figure 13:**
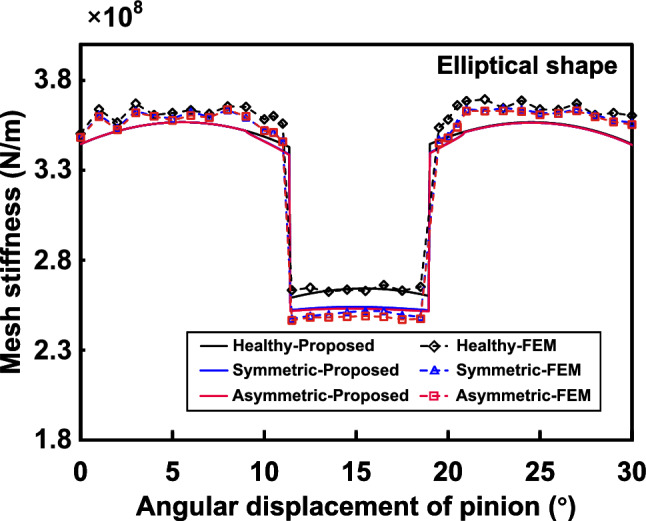
Comparison of the TVMS under symmetric and asymmetric elliptical spalling evaluated by proposed method and FEM.

**Figure 14:**
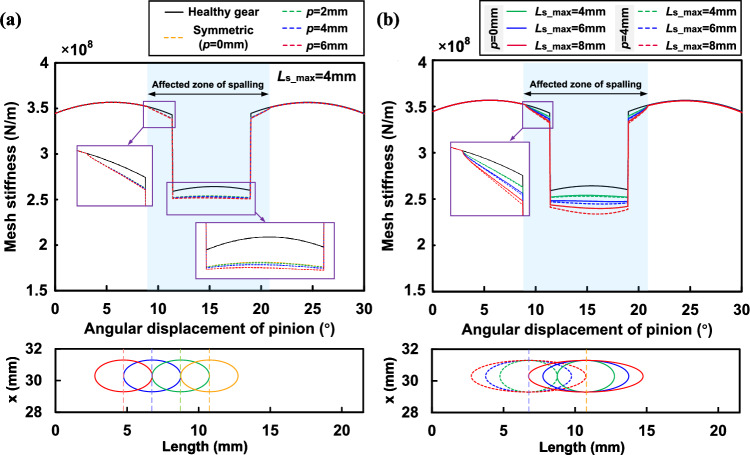
TVMS of proposed method for elliptical spalling with different offsets and maximum spalling lengths.

The TVMS evaluated by the proposed method and FEM with a 4*mm* offset of the elliptical center are shown in Fig. 13. Compared to the sudden change in TVMS caused by rectangular spalling, elliptical spalling shows a steady decrease in TVMS during the process of entering the spalling zone from the normal engagement zone. This is mainly influenced by the variation in spalling length. The reduction in TVMS gradually increases until it reaches a maximum at the long axis of the ellipse and then gradually decreases until it leaves the spalling zone.

The effects of offset and maximum length on the TVMS are further discussed. First, the TVMS of gears with elliptical spalling under the offsets of 2*mm*, 4*mm* and 6*mm* are obtained, as shown in Fig. 14a. It can be seen that the offset effect of the defects is not significant at the current spalling size for both single and double tooth meshing zones. Only when the offset is large (6*mm* in this case) can the effect of the asymmetric spalling be seen near the position of the long axis. This also shows that the asymmetric effect can be disregarded for a solitary, minor elliptical spalling. Figure 14b shows the TVMS for elliptical spalling with maximum lengths $L_{s\_max}$ of 4*mm*, 6*mm*, 8*mm* under symmetric ($p=0mm$) and asymmetric ($p=4mm$) conditions. It is evident that the impact of asymmetric spalling increases as the maximum length grows. Furthermore, the combination of maximum length and offset has a compounding effect on the TVMS trajectory, resulting in a tendency to speed up. This variance would have a substantial impact on the deterioration characteristics of the dynamic response of the spalled gear.

**Figure 15:**
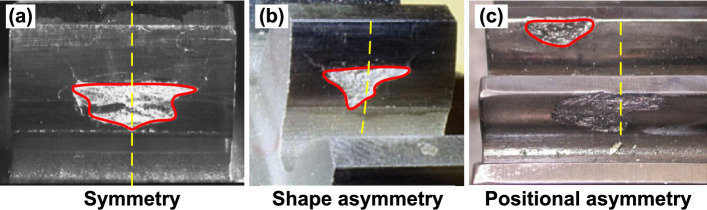
Asymmetric differences in irregular shapes^25^.

**Figure 16:**
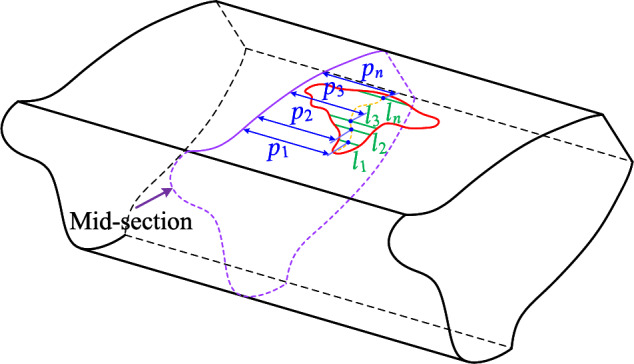
Geometric modelling of irregular shapes with two parameters.

### Case 3: irregular shape

As shown in Fig. 15, spalling defects localised in real gear teeth often appear as irregular shapes. Modelling irregular shapes is a more intricate task than regular shapes like rectangles and ellipses. On one hand, the irregular variation of spalling length along the *x-*coordinates poses a challenge in accurately describing it. On the other hand, the spalling performs both shape asymmetry (see Fig. 15b) and positional asymmetry (see Fig. 15c). The shape and location of irregular spalling can be determined by two essential parameters: the spalling length, $L_s(x)$, for any *x*-coordinate, and the distance, *p*(*x*), from the gear mid-section to the centre of the spalling length. An approximate description of the spalling’s properties can be obtained through discrete sampling and polynomial fitting of these two parameters. As given in Fig. 16, $\left\{ l_1,l_2,l_3,...,l_n \right\}$ and $\left\{ p_1,p_2,p_3,...,p_n \right\}$ are the discrete lengths and offsets of the tooth spalling at different *x*. The continuous spalling length function $L_s(x)$ and offset function *p*(*x*) can be separately approximated in polynomial functions:

30 $$\begin{aligned} L_s\left( x \right)= & {} a_nx^n+a_{n-1}x^{n-1}+\cdots +a_2x^2+a_1x+a_0, x\in \left[ x_{s\_st},x_{s\_end} \right] , \end{aligned}$$

31 $$\begin{aligned} p\left( x \right)= & {} b_nx^n+b_{n-1}x^{n-1}+\cdots +b_2x^2+b_1x+b_0, x\in \left[ x_{s\_st},x_{s\_end} \right] , \end{aligned}$$

where $a_0$-$a_n$, $b_0$-$b_n$ are constants of the fitted function, which can be obtained in Matlab using the ’*polyfit*’ function.

**Figure 17:**
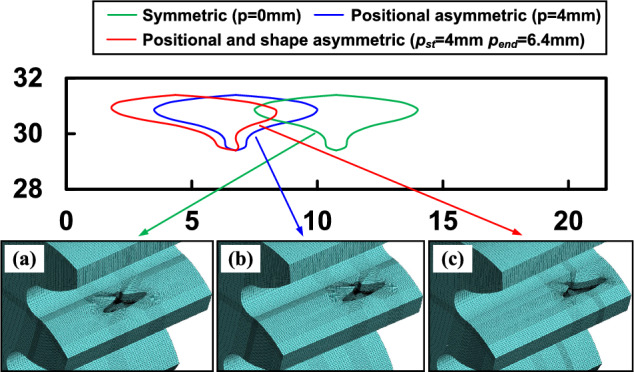
Fitted curves and FEM of different irregular shapes.

In this case, we will examine two scenarios: one where the irregular spalling is symmetrical in shape but off-centre, and the other where both shape and position are asymmetrical. Figure 17 presents the fitted curves of the irregular shape, alongside the corresponding finite element model for both types of cases mentioned previously. The starting and ending points of the spalling are set to $x_{s\_st}=29.395mm$ and $x_{s\_end}=31.395mm$, while the depth $h_s$ is assumed to be fixed at 0.9*mm*. For comparison, this case sets the offset *p*(*x*) of the spalling centreline from the gear mid-section to be constant at 4*mm* when only positional asymmetry is considered. When taking into account both positional and shape asymmetry, the offset at the starting point of the spalling to be 4*mm* and increase it linearly until it reaches 6.4*mm* at the ending point. Therefore, the defect ratio models incorporate the approximate spalling length function and the offset function. The TVMS under arbitrary irregular spalling can be calculated from the equations in “TVMS formula for gears with asymmetric spalling defects” section.

**Figure 18:**
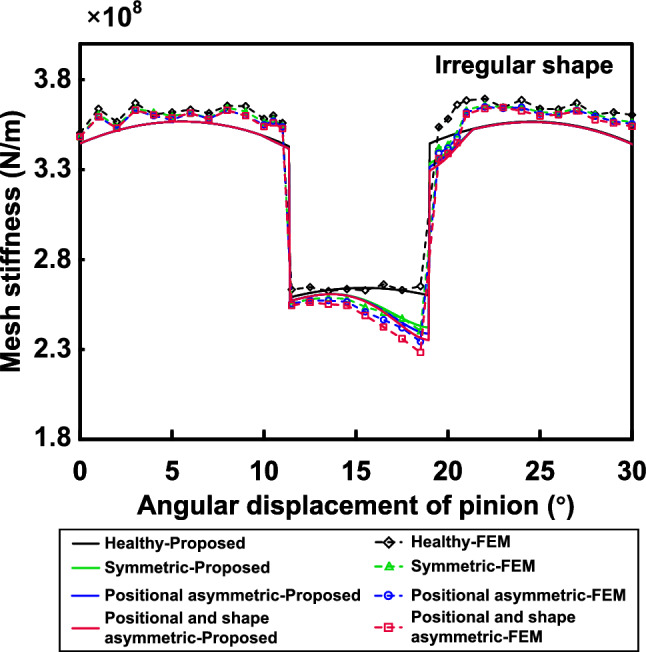
Comparison of the TVMS under symmetric and asymmetric irregular spalling evaluated by proposed method and FEM.

Firstly, we will solely focus on the positional asymmetry condition. The FEM and the proposed model present a similar trend in describing irregular spalling for TVMS, as depicted in Fig. 18. Although there is some deviation between the results obtained by the two methods due to uncertainty in the simulation setup, the deviation falls within an acceptable range. Comparison with the healthy condition shows that the change trajectory of TVMS under irregular spalling is characterised by irregularity. A further comparison of the spalling length changes reveals that the spalling length has a crucial impact on TVMS degradation. However, the irregular spalling offset also results in further degradation of TVMS. In the case of longer spalling length, TVMS degradation is notably more significant. Next, we will investigate the combined effects of positional and shape asymmetry. Compared to the shape-symmetric state, although the spalling length remains constant for the same *x*-coordinate, the impact of torsional stiffness becomes increasingly pronounced as the offset increases, exacerbating the overall stiffness reduction further. The features outlined above are present in both the FEM and the proposed model.

**Figure 19:**
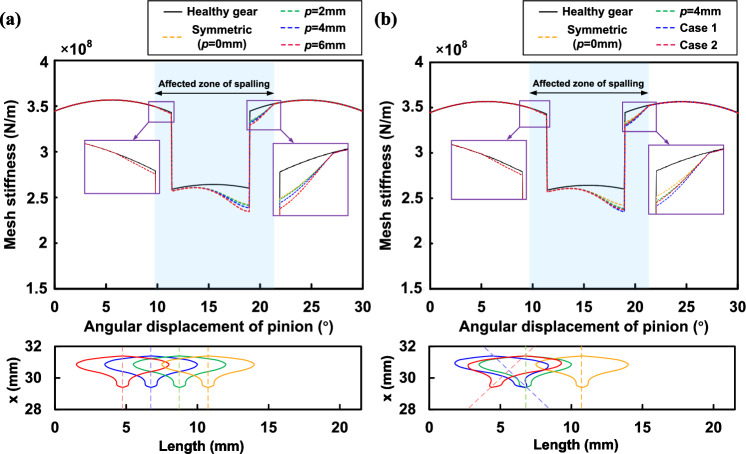
TVMS of proposed method for irregular spalling with different offsets.

Next, we will explore the impact of various offsets on TVMS. For the condition of shape symmetry, we set the same spalling defects to be located at offsets of 2*mm*, 4*mm* and 6*mm*, respectively, and calculate the TVMS using the proposed model. The results can be seen in Fig. 19a. Through the comparison, it is evident that the TVMS trajectories alter similarly at various offsets owing to the spalling’s irregular shape. As the spalling length increases, the degradation of the TVMS with offset becomes more significant, particularly in the single-tooth meshing region. The analysis of positional asymmetry is subsequently carried out. Two categories of irregular spalling conditions are set up. Case 1 represents the previous state with a start point offset of 4*mm* and an end point offset of 6.4*mm*. Conversely, Case 2 denotes the opposite scenario. Additionally, constant offsets of 0*mm* (symmetric spalling) and 4*mm* are introduced for comparison. It is evident that the asymmetry caused by the offset results in an inconsistent TVMS decline rate, as opposed to the symmetric shape of the consistent decline trajectory. The offset significantly affects the decline of TVMS particularly at longer spalling length condition. In this regard, the superimposed effect of shape and position asymmetry should not be neglected.

## Conclusion

This article proposed an improved TVMS model of a spur gear pair considering asymmetric spalling defects. Compared to the traditional TVMS model, the introduction of the torsional stiffness in the improved TVMS model provides a more complete consideration of the axial defects position. Additionally, a generic shape-independent TVMS calculation method and an improved FEM validation method are developed, which enable TVMS evaluation with different types of spalling shapes under asymmetric condition. The main conclusions are summarized below.

1. Validated by FEM, the improved TVMS model proposed in this paper is accurate enough to describe the effects of spalling at different locations. The TVMS for various spalling shapes can be well evaluated.
2. Whether the shape is regular shapes (such as rectangles or ellipses) or irregular, asymmetric spalling causes more reductions in TVMS than the same spalling under symmetric conditions. And the larger the offset, the more significant this effect is.
3. The size of the spalling defects positively affects the asymmetry effect and the total stiffness. When the spalling defect is small, the torsional stiffness caused by the asymmetric effect is not large, and its effect on the total stiffness is not significant. However, as the spalling length increases, its large offset will exacerbate the rapid reduction of total stiffness, and the asymmetric effect will become a non-negligible problem.
4. The length and offset of the spalling defects have a superimposed effect on the change in TVMS. The reduction in TVMS is primarily determined by the spalling length, and the asymmetric effect further accelerates the TVMS change in the longer regions within the spalling defect. It makes the spalling defects with the same spalling length show different stiffness trajectories at different offsets, and further leads to the change in vibration characteristics.

There are still several questions to answer in future:

1. This paper considers the effect of asymmetric effects on TVMS. Further exploration of the effect on dynamic response characteristics with the aid of multi-degree-of-freedom dynamics modelling will be considered as our future work.
2. In addition, the impact of uneven loading of the contact force and asymmetric spalling depth variations on the TVMS and the dynamic response will be investigated as part of future research.
3. It is necessary to consider that the effective loading area of the gear mesh point located in the non-spalled region may not be the entire healthy gear tooth cross-section due to the influence of spalling defects. This requires further justification.

## Data Availability

The datasets used and/or analysed during the current study available from the corresponding author on reasonable request.
